# Supplementary material for: Red-shifting mutation of light-driven sodium-pump rhodopsin
Source: Nat Commun. 2019 Apr 30;10:1993. doi: 10.1038/s41467-019-10000-x (PMC6491443; doi:10.1038/s41467-019-10000-x)
Supplement: Supplementary file 1 — Supplementary Information [file 41467_2019_10000_MOESM1_ESM.pdf]

Supporting Information:

Red-shifting Mutation of Light-driven Sodium Pump Rhodopsin

Inoue et al.

Supplementary Table 1. **Observed vs computed vertical excitation energies ΔE_{S1-S0} (kcal/mol) and maximum absorption wavelengths (λ_{max}) for KR2 WT and its mutants P219G, P219T, S254A, and P219T/S254A; ARM models computed at CASPT2//CASSCF(12,12)/6-31G*/AMBER level of theory.** The oscillator strength (f_{Osc}) for $S_0 \rightarrow S_1$ and $S_0 \rightarrow S_2$ transitions and standard deviation (DEV.ST) are also shown.

Sample	Obs. ΔE_{S1-S0} (λ_{max})	Comp. ΔE_{S1-S0} (λ_{max})	$f_{Osc\ S0-S1}$	$f_{Osc\ S0-S2}$	Error	DES.ST
**KR2 WT**	54.4 (525)	55.2 (517)	1.06	0.43	0.8	0.2415
**P219G**	53.4 (535)	54.3 (526)	1.01	0.40	0.9	0.1838
**P219T**	52.7 (542)	53.5 (534)	1.05	0.42	0.8	0.4423
**S254A**	52.4 (545)	53.1 (538)	1.00	0.41	0.7	0.3838
**P219T/S254A**	50.6 (565)	51.5 (555)	1.12	0.46	0.9	0.5638

Supplementary Table 2. Computed 3-root CASPT2//CASSCF(12,12)/6-31G*/AMBER vertical excitation energies ΔE_{S1-S0} (kcal/mol) of KR2 WT and P219G, P219T, S254A, P219T/S254A mutants, computed when the amino acids where turned off individually and also concurrently.

Sample	Computed E_{S0} (a.u.)	Computed E_{S1} (a.u.)	ΔE_{S1-S0} (kcal/mol)	ΔE_{off} (kcal/mol)	$\Delta\Delta E = \Delta E_{S1-S0} - \Delta E_{off}$ (kcal/mol)
**KR2 WT**	-872.002324	-871.914423	55.2		
P219 OFF	-872.005518	-871.919671		53.9	+1.3
S254 OFF	-871.993035	-871.909239		52.6	+2.6
Both OFF	-871.984483	-871.901750		51.9	+3.2
**P219G**	-872.004081	-871.917494	54.3		
G219 OFF	-872.000409	-871.910690		56.3	-2.0
S254 OFF	-872.001467	-871.918690		51.9	+2.4
Both OFF	-871.987028	-871.896631		56.7	-2.4
**P219T**	-872.004267	-871.919068	53.5		
T219 OFF	-872.000397	-871.911704		55.7	-2.2
S254 OFF	-872.001308	-871.920153		50.9	+2.5
Both OFF	-871.986901	-871.897137		56.3	-2.9
**S254A**	-871.998014	-871.913387	53.1		
P219 OFF	-872.010538	-871.930712		50.1	+3.0
A254 OFF	-872.019812	-871.928719		57.2	-4.1
Both OFF	-872.020781	-871.929141		57.5	-4.4
**P219T/S254A**	-871.999028	-871.917009	51.5		
T219 OFF	-871.990471	-871.904659		53.8	-2.4
A254 OFF	-872.045650	-871.959600		54.0	-2.5
Both OFF	-872.045994	-871.953091		58.3	-6.8

Supplementary Table 3. Double and single bond lengths (Å) of QM/MM models built with ARM protocol at CASSCF(12,12)/6-31G*/AMBER level of theory of KR2 WT and its mutants.

Bond/Sample	WT	P219T	S254A	P219T/S254A
C5=C6	1.37	1.371	1.371	1.375
C6-C7	1.486	1.484	1.483	1.481
C7=C8	1.355	1.356	1.355	1.356
C8-C9	1.475	1.473	1.472	1.473
C9=C10	1.362	1.362	1.362	1.365
C10-C11	1.456	1.450	1.453	1.451
C11=C12	1.355	1.357	1.357	1.360
C12-C13	1.462	1.461	1.464	1.465
C13=C14	1.362	1.364	1.368	1.369
C14-C15	1.445	1.442	1.439	1.439
C15=N	1.289	1.288	1.288	1.290

Supplementary Table 4. Double and single bond lengths (Å) of QM/MM models built with ARM protocol at CASSCF(12,12)/6-31G*/AMBER level of theory of KR2 WT and its mutants.

Dihedral/Sample	WT	P219T	S254A	P219T/S254A
C4-C5=C6-C7	172.46	171.92	172.50	171.49
C5=C6-C7=C8	-175.74	-174.40	-174.26	-175.44
C6-C7=C8-C9	173.97	173.45	173.98	173.14
C7=C8-C9=C10	-172.54	-173.27	-172.04	-173.40
C8-C9=C10-C11	170.81	170.76	171.61	170.12
C9=C10-C11=C12	-178.55	-177.45	-178.93	-177.27
C10-C11=C12-C13	162.81	162.35	161.32	160.18
C11=C12-C13=C14	-171.66	-172.00	-170.36	-169.90
C12-C13=C14-C15	151.20	151.39	150.02	149.41
C13=C14-C15=N	-178.92	-177.98	-176.10	-174.46
C14-C15=N-C_δ	159.55	159.86	157.89	152.10

Supplementary Table 5. **Total positive charge on the C11H-C12H-C13(Me)-C14H-C15H-NHR moiety (i.e., relevant to the C11=C12 isomerization).** In *parenthesis* we also give the charges corresponding to the shorter C13(Me)-C14H-C15H-NHR moiety (i.e., relevant to the C13=C14 isomerization).

Protein	S_0 Charge (*e*)	S_1 Charge (*e*)	S_2 Charge (*e*)
KR2 WT	0.83 *(0.78)*	0.45 *(0.42)*	0.74 *(0.70)*
P219G	0.85 *(0.80)*	0.42 *(0.39)*	0.76 *(0.72)*
P219T	0.84 *(0.78)*	0.44 *(0.40)*	0.74 *(0.70)*
S254A	0.87 *(0.81)*	0.41 *(0.37)*	0.79 *(0.73)*
P219T/S254A	0.81 *(0.77)*	0.47 *(0.45)*	0.72 *(0.69)*

Supplementary Table 6. **List of primers used for the site-directed mutations.**

Mutation	Sense primer	Anti-sense primer
KR2 L168Y	CCTGACTGCGTTTTATGTCTGG GGAGCCATTAG	CTAATGGCTCCCCAGACATAAA ACGCAGTCAGG
KR2 G171S	GCGTTTCTGGTCTGGAGCGCCA TTAGCAGTGCG	CGCACTGCTAATGGCGCTCCAG ACCAGAAACGC
KR2 F178Y	CATTAGCAGTGCGTTCTATTTTC ACATTCTCTGG	CCAGAGAATGTGAAAATAGAA CGCACTGCTAATG
KR2 G146S	CAGTTTTGGTTCTCAAGCGCCA TGATGATCATTAC	GTAATGATCATCATGGCGCTTG AGAACCAAAACTG
KR2 M149S	GTTCTCAGGTGCCATGAGCATC ATTACAGGC	GCCTGTAATGATGCTCATGGCA CCTGAGAAC
KR2 I150T	CTCAGGTGCCATGATGACCATT ACAGGCTATATC	GATATAGCCTGTAATGGTCATC ATGGCACCTGAG
KR2 G153S	GCCATGATGATCATTACAAGCT ATATCGGACAG	CTGTCCGATATAGCTTGTAATG ATCATCATGGC
KR2 G156S	CATTACAGGCTATATCAGCCAG TTTTACGAAGTG	CACTTCGTAAAACTGGCTGATA TAGCCTGTAATG
KR2 I181T	GCGTTCTTCTTTCACACCCTCTG GGTTATGAAG	CTTCATAACCCAGAGGGTGTGA AAGAAGAACGC
KR2 F211Y	CAACATCTGGATTCTGTATCTG ATCTCTTGGACG	CGTCCAAGAGATCAGATACAGA ATCCAGATGTTG
KR2 W215Y	CTGTTTCTGATCTCTTATACGTT GTACCCAGGTG	CACCTGGGTACAACGTATAAGA GATCAGAAACAG
KR2 P219T	CTCTTGGACGTTGTACACCGGT GCGTATTTAATG	CATTAAATACGCACCGGTGTAC AACGTCCAAGAG
KR2 P219G	CTCTTGGACGTTGTACGGCGGT GCGTATTTAATG	CATTAAATACGCACCGCCGTAC AACGTCCAAGAG
KR2 S254A	TGCAGATGTGTCGGCGAAAGTC ATTTATG	CATAAATGACTTTCGCCGACAC ATCTGCA
JsNaR G216P	CACCTGGACCATTTACCCGCTG GTGTATCTGGTTC	GAACCAGATACACCAGCGGGT AAATGGTCCAGGTG
JsNaR S247A	CTTTGCGGACGTTACCGCGAAA GCGGTGTTCGGC	GCCGAACACCGCTTTCGCGGTA ACGTCCGCAAAG
NpHR P226T	CATGTGGCTCGGCTACACCATC GTGTGGGCACTC	GAGTGCCCACACGATGGTGTAG CCGAGCCACATG
PoXeR P179T	GTTTTGGGTTAGCTATACCCTG GTTTGGCTGATC	GATCAGCCAAACCAGGGTATAG CTAACCCAAAAC

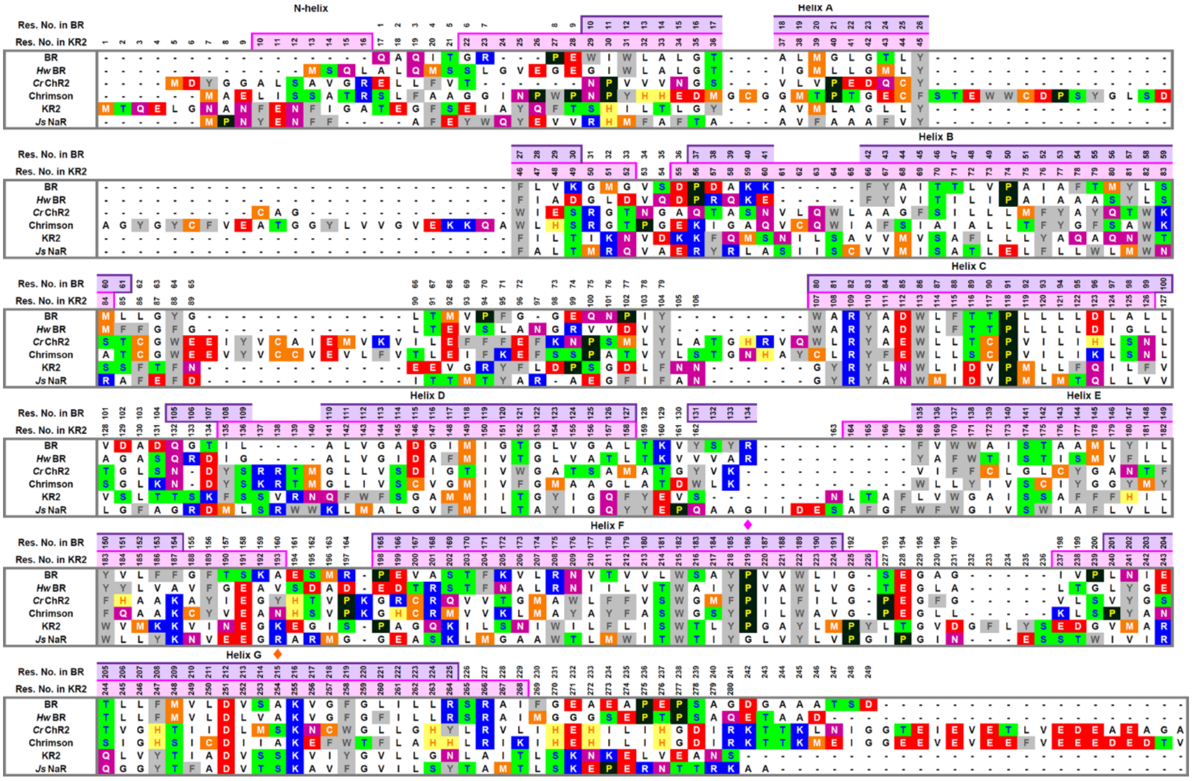

Supplementary Figure 1. Multiple alignment of amino acid sequences of microbial rhodopsins. The amino acid sequences were aligned by ClustalW[1] for BR, BR of *Haloquadratum walsbyi* (*Hw*BR), ChR2 without the C-terminal sequence, Chrimson, KR2 and *Js*NaR. The residue numbers (Res. No.) in BR and KR2 are shown in the first and second row, respectively. Transmembrane helices of BR and KR2 were indicated by purple and pink rectangles, respectively, according to their X-ray crystallographic structures ((PDB code: 1M0L (BR)[2] and 3X3C (KR2)[3]). The positions of KR2 Pro219 and Ser254 are indicated by pink and orange diamonds, respectively.

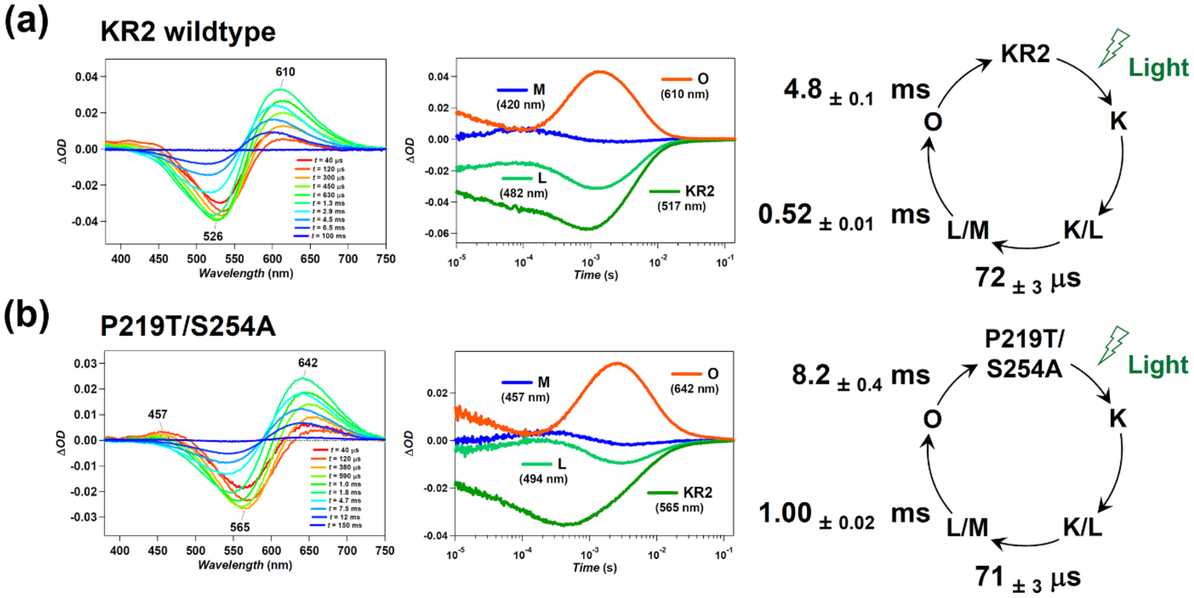

Supplementary Figure 2. The photocycles of KR2 mutants. Transient absorption spectra (left), the time evolutions of transient absorption change at specific wavelengths (middle) and the photocycles determined by the curve fitting with multi-exponential functions (right) of KR2 WT (a) and P219T/S254A (b), in POPE/POPG liposome (molar ratio = 3 : 1) with a protein-to-lipid molar ratio of 1:50. The lifetimes and their standard deviations of the photo-intermediates in are indicated in the photocycles. The green, light-green, blue, and orange lines in (b) show the photo-bleaching of the initial sate, accumulations of L-, M- and O-intermediates, respectively. Source data are provided as a Source Data file

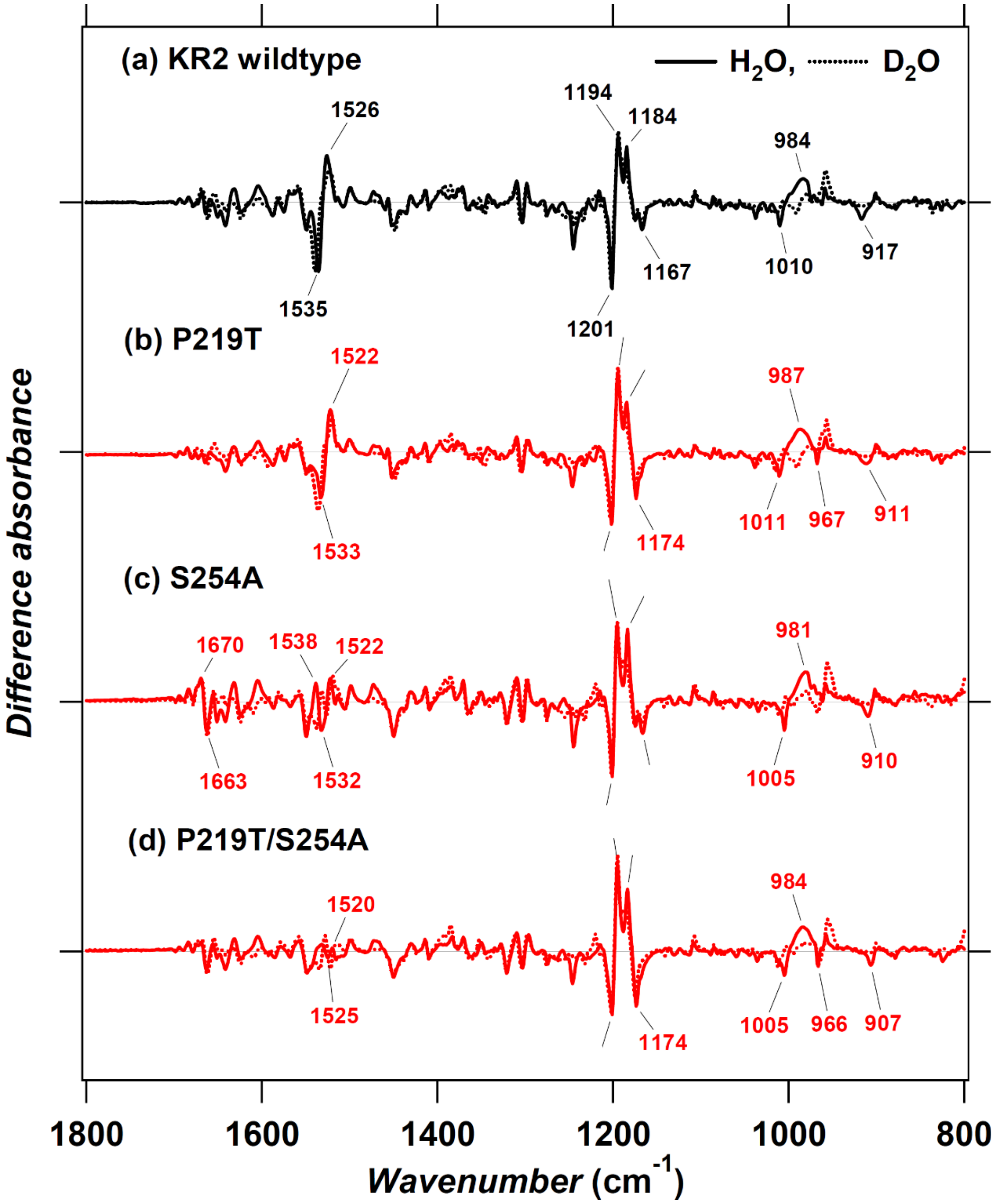

Supplementary Figure 3. Light-induced infrared absorption changes of KR2 mutants. Light-induced FTIR difference spectra of (a) KR2 WT (black) and the mutants (red), (b) P219T, (c) S254A and (d) P219T/S254A, in the 1800-800 cm^{-1} region at $T = 77$ K and pH 8.0. Solid and dotted lines represent the samples hydrated with H_2O and D_2O, respectively. Source data are provided as a Source Data file

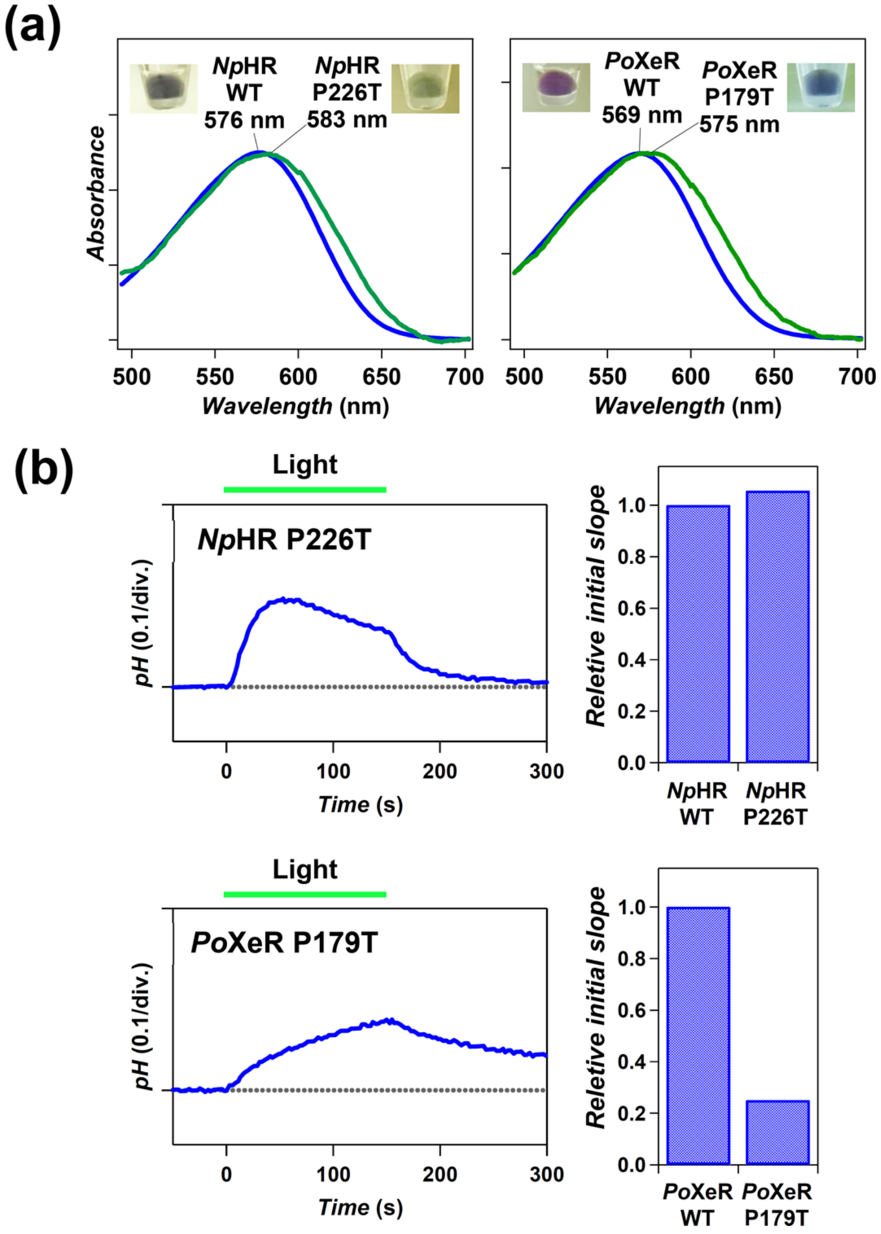

Supplementary Figure 4. The effect of mutation at the proline corresponding to KR2 Pro219 in the chloride pump (*Np*HR) and inward proton pump (*Po*XeR) rhodopsins. (a) Absorption spectra of *Np*HR WT, P226T, *Po*XeR WT and P179T. The wildtype and mutant proteins are indicated in blue and green, respectively. (b) Ion pump activity assays by monitoring pH changes in the external media of the suspension of *E. coli* cells expressing *Po*XeR P179T and *Np*HR P226T (left) and the relative initial slopes of each to the wildtype (right). The light was illuminated at t = 0-150 s indicated by light-green bars. Source data are provided as a Source Data file

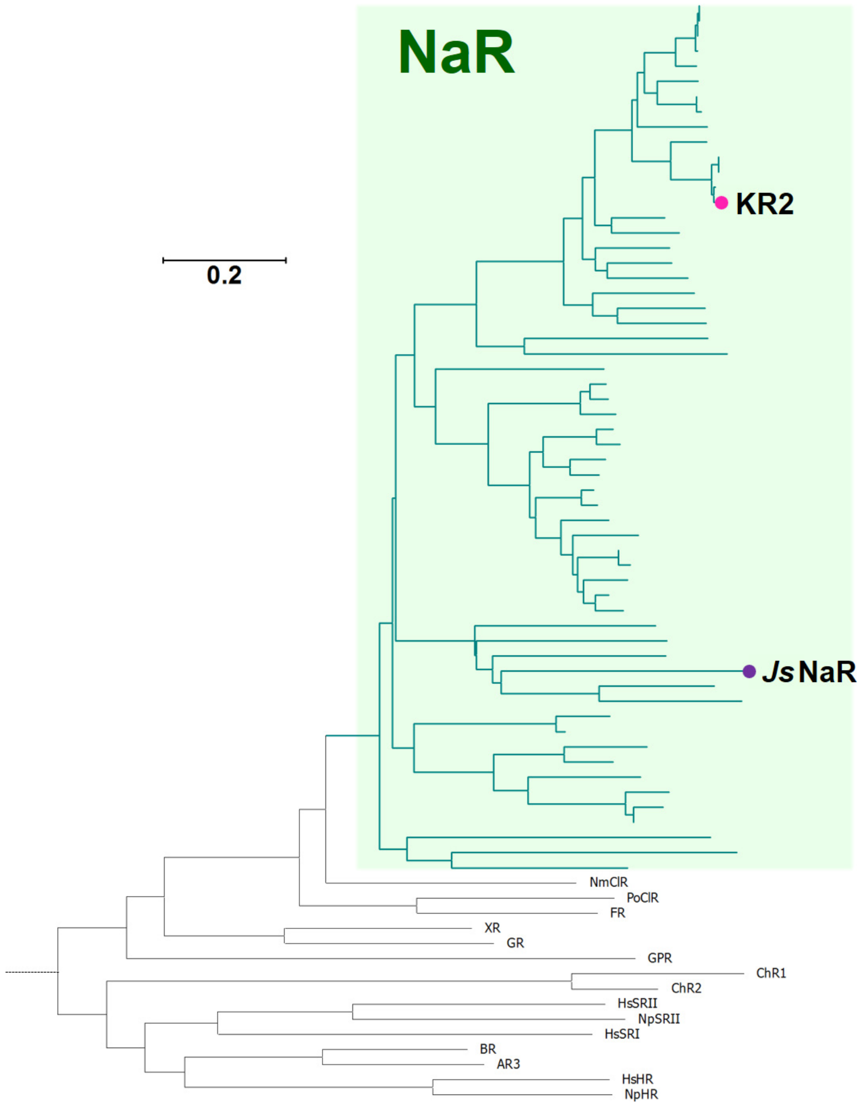

Supplementary Figure 5. Phylogenetic relationship of KR2 and *Js*NaR in the microbial rhodopsin sub-family. NaRs are indicated with green branches. The evolutionary history was inferred using the Neighbor-Joining method[4]. The optimal tree with the sum of branch length = 17.62164409 is shown. The tree is drawn to scale, with branch lengths in the same units as those of the evolutionary distances used to infer the phylogenetic tree. The evolutionary distances were computed using the Poisson correction method[5] and are in the units of the number of amino acid substitutions per site. The analysis involved 73 amino acid sequences. All ambiguous positions were removed for each sequence pair. There were a total of 393 positions in the final dataset. Evolutionary analyses were conducted in MEGA6[6]. KR2 and *Js*NaR are indicated by pink and purple circles, respectively.

Supplementary References

1 Thompson, J. D., Higgins, D. G. & Gibson, T. J. Clustal-W - Improving the Sensitivity of Progressive Multiple Sequence Alignment through Sequence Weighting, Position-Specific Gap Penalties and Weight Matrix Choice. *Nucleic Acids Res.* **22**, 4673-4680, (1994).

2 Schobert, B., Cupp-Vickery, J., Hornak, V., Smith, S. & Lanyi, J. Crystallographic structure of the K intermediate of bacteriorhodopsin: conservation of free energy after photoisomerization of the retinal. *J. Mol. Biol.* **321**, 715-726, (2002).

3 Kato, H. E. *et al.* Structural basis for Na^+ transport mechanism by a light-driven Na^+ pump. *Nature* **521**, 48-53, (2015).

4 Saitou, N. & Nei, M. The neighbor-joining method: a new method for reconstructing phylogenetic trees. *Mol. Biol. Evol.* **4**, 406-425, (1987).

5 Zuckerkandl, E. & Pauling, L. in *Evolving Genes and Proteins* (eds V. Bryson & H. J. Vogel) 97-166 (Academic Press, New York 1965).

6 Tamura, K. *et al.* MEGA5: molecular evolutionary genetics analysis using maximum likelihood, evolutionary distance, and maximum parsimony methods. *Mol. Biol. Evol.* **28**, 2731-2739, (2011).
